# Important amino acid residues of hexachlorocyclohexane dehydrochlorinases (LinA) for enantioselective transformation of hexachlorocyclohexane isomers

**DOI:** 10.1007/s10532-017-9786-9

**Published:** 2017-03-01

**Authors:** Nidhi Shrivastava, Ankit S. Macwan, Hans-Peter E. Kohler, Ashwani Kumar

**Affiliations:** 1 Academy of Scientific and Innovative Research, New Delhi, India; 2 Environmental Biotechnology Section, CSIR-Indian Institute of Toxicology Research, Lucknow, India; 3 Department of Clinical and Experimental Medicine, Linköping University, Linköping, Sweden; 4 Department of Environmental Microbiology, Swiss Federal Institute for Aquatic Science and Technology (Eawag), Dübendorf, Switzerland

**Keywords:** HCH dehydrochlorinase LinA, Enantioselectivity, α-HCH enantiomers

## Abstract

LinA-type1 and LinA-type2 are two well-characterized variants of the enzyme ‘hexachlorocyclohexane (HCH)-dehydrochlorinase’. They differ from each other at ten amino acid positions and exhibit differing enantioselectivity for the transformation of the (–) and (+) enantiomers of α-HCH. Amino acids responsible for this enantioselectivity, however, are not known. An in silico docking analysis identified four amino acids (K20, L96, A131, and T133) in LinA-type1 that could be involved in selective binding of the substrates. Experimental studies with constructed mutant enzymes revealed that a combined presence of three amino acid changes in LinA-type1, i.e. K20Q, L96C, and A131G, caused a reversal in its preference from the (–) to the (+) enantiomer of α-HCH. This preference was enhanced by the additional amino acid change T133 M. Presence of these four changes also caused the reversal of enantioselectivity of LinA-type1 for δ-HCH, and β-, γ-, and δ-pentachlorocyclohexens. Thus, the residues K20, L96, A131, and T133 in LinA-type1 and the residues Q20, C96, G131, and M133 in LinA-type 2 appear to be important determinants for the enantioselectivity of LinA enzymes.

**Electronic supplementary material:**

The online version of this article (doi:10.1007/s10532-017-9786-9) contains supplementary material, which is available to authorized users.

## Introduction

Technical hexachlorocyclohexane (t-HCH) and lindane, the purified γ-HCH isomer that is also the insecticidal agent, are organochlorine insecticides that were extensively used throughout the world between the 1940s and the 1990s for controlling a wide range of agricultural and public health pests (Lal et al. 2010). Residue issues from their production and usage still pose severe environmental problems and led to the addition of α-HCH, β-HCH, and γ-HCH to the list of persistent organic pollutants (POPs) under the Stockholm Convention for Protecting Human Health and the Environment from Persistent Organic Pollutants in 2009 (UNEP 2009).

Aerobic transformation of HCH isomers, especially the transformation of γ-HCH, has been studied in some detail (Lal et al. 2010; Nagata et al. 2007; Raina et al. 2008). Microbial degradation of γ-HCH under aerobic conditions starts with two dehydrochlorination reactions catalyzed by the enzyme HCH-dehydrochlorinase (LinA) (Lal et al. 2010; Nagata et al. 2007). First, γ-HCH is dehydrochlorinated to γ-pentachlorocyclohexene (γ-PCCH), which is then further dehydrochlorinted to the putative and unstable intermediate 1,3(*R*),4,6(*R*)-tetrachlorocyclohexa-1,4-diene (TCDN) (Trantírek et al. 2001). In the course of microbial metabolism of γ-HCH, TCDN is metabolized by LinB in two steps to 2,5-dichloro-2,5-cyclohexadiene-1,4-diol (Lal et al. 2010; Nagata et al. 2007). However, in enzymatic assays when LinA is incubated with γ-HCH as the substrate without the presence of LinB, γ-HCH is first converted to γ-PCCH and then to trichlorobenzenes presumably via TCDN (Geueke et al. 2013; Lal et al. 2010). LinA also mediates the transformation of α- and δ-HCH to the corresponding PCCHs and subsequently to TCBs in in vitro enzymatic assays (Geueke et al. 2013; Suar et al. 2005).

**Fig. 1:**
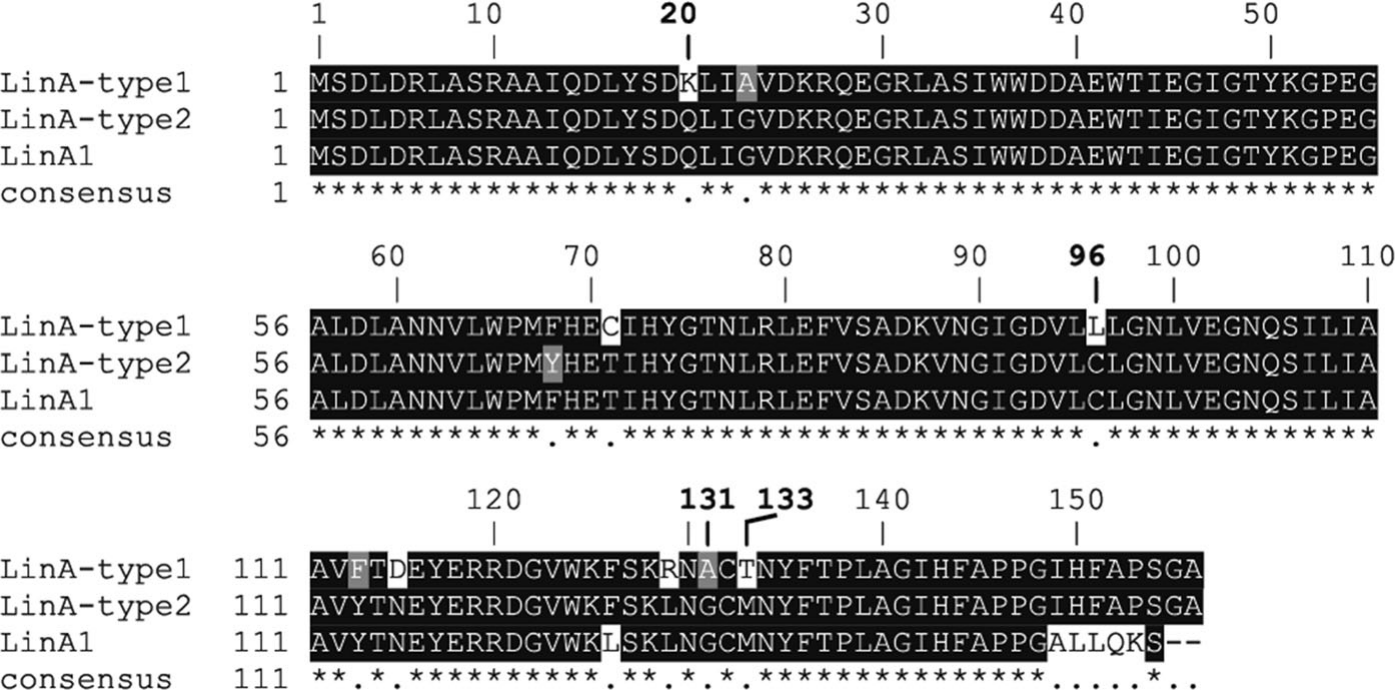
Alignment and sequence differences among the LinA variants investigated and discussed in this study. *Bold numbers* indicate the amino acids that were mutated in LinA-type 1 for assessing enantioselectivity with regard to the transformation of α-HCH

LinA-type1, LinA-type2, and LinA1 are three well-characterized variants of the enzyme ‘hexachlorocyclohexane (HCH)-dehydrochlorinase (LinA)’. LinA-type1 was initially characterized from the HCH degrading strain *Sphingobium japonicum* UT26 as LinA (Imai et al. 1991) and was subsequently shown to be identical to LinA2 from *Sphingobium indicum* B90A (Lal et al. 2010). The gene of LinA-type2 was obtained from the metagenome of an HCH-contaminated site (Macwan et al. 2011) and LinA1 was identified in *Sphingobium indicum* B90A (Kumari et al. 2002; Lal et al. 2010). Sequence alignments and sequence differences among the three LinA variants are shown in Fig. 1.

Enantioselectivity of LinA variants for the transformation of various HCH-isomers and the transformation of the formed PCCHs is well established (Geueke et al. 2013; Macwan et al. 2012; Suar et al. 2005; Trantírek et al. 2001). LinA-type1 turned out to mediate the transformation of γ-HCH to the single PCCH enantiomer (3*R*,4*S*,5*S*,6*R*)-1,3,4,5,6-PCCH (γ-PCCH-2) (Trantírek et al. 2001). Furthermore, LinA-type1 enantioselectively acted on racemic γ-PCCH, whereby transformation of γ-PCCH-2 was substantially faster than transformation of (3*S*,4*R*,5*R*,6*S*)-1,3,4,5,6-PCCH (γ-PCCH-1). The authors proposed that the enantioselective formation of γ-PCCH-2 by LinA-type1 was due to the ability of the enzyme to differentiate the enantiotopic pairs of vicinal HCl present in γ-HCH (Trantírek et al. 2001).

Like γ-HCH, δ-HCH is achiral but can be metabolized to two chiral δ-PCCH enantiomers depending on which of the two enantiotopic trans-diaxial HCl pairs is eliminated (Geueke et al. 2013). Enantioselective formation of δ-PCCH-2 in an enantiomeric excess of ~98 and ~87% has been reported for the reaction of δ-HCH with LinA-type1 and LinA1, respectively. Enantioselectivity was also shown for transformation of the formed δ-PCCH, whereby LinA-type1 and LinA1 exhibited preference for the transformation of δ-PCCH-2 and δ-PCCH-1, respectively (Geueke et al. 2013).

In contrast to γ-HCH and δ-HCH, α-HCH is chiral and occurs as a racemate in technical-HCH. Recombinant *E. coli* cells expressing a functional LinA-type1 *S*-glutathione transferase fusion protein mediated preferential transformation of the (–) enantiomer of α-HCH and the reaction was accompanied by the enantioselective formation of (3*R*,4*R*,5*S*,6*S*)-1,3,4,5,6-PCCH (β-PCCH-2) over the formation of (3*S*,4*S*,5*R*,6*R*)-1,3,4,5,6-PCCH (β-PCCH-1) (Suar et al. 2005). Interestingly, *E. coli* cells that were expressing a functional LinA1 *S*-glutathione transferase fusion protein preferentially transformed the (+) enantiomer of α-HCH (Suar et al. 2005). The two enzymes also showed differing enantioselectivity for the transformation of β-PCCH. LinA-type1 preferentially transformed β-PCCH-1 while LinA1 preferentially transformed β-PCCH-2 (Suar et al. 2005).

LinA1 differs from LinA-type1 only at eleven amino acid positions in the 1–148 amino acid but strongly differs in the remaining downstream region (Fig. 1) due to insertion of an *IS*6100 element at the 3′ end of its gene (Lal et al. 2010). The number of amino acid residues in LinA-type1 and LinA1 are 156 and 154, respectively, and the overall identity between the two proteins is 88% (Fig. 1). However, it is not clear at present which amino acid(s) are responsible for the reversed enantioselectivity of the two enzymes.

The primary structure of LinA-type 2 differs only in one amino acid (F126L) from LinA1 in the 1–148 amino acid region and the remaining 149–156 region is identical to the corresponding region of LinA-type1 (Fig. 1). As both LinA1 and LinA-type2 exhibit preference for the transformation of the (+) enantiomer of α-HCH (Macwan et al. 2012), we expect that amino acids in the 1–148 amino acid region and not those in the 149–153 region determine enantioselectivity of LinA-type1, LinA-type2, and LinA1.

Crystal structures of both LinA-type1 (Okai et al. 2010) and LinA-type2 (Macwan et al. 2012) have been determined. Docking experiments suggested that within the 1–148 amino acid region the diverging residues 20 (K, C) 96 (L, C), 131 (A, G), and 133 (T, M), vicinal to the active site, might play a role in substrate binding and, therefore, in determining enantioselectivity of LinA-type1 and LinA-type2 (Macwan et al. 2012).

To further elucidate the role of these residues, we overexpressed eight mutant LinA-type1 enzymes with one to four of the above amino acids changes. Measurement of their specificity constants *k*_cat_/*K*_m_, and enantioselectivities revealed that not one single divergent amino acid determined enantioselectivity, but that the cumulative effect of the four diverging residues 20 (K, C) 96 (L, C), 131 (A, G), and 133 (T, M) was needed for the opposite enantioselectivity of LinA-type1 and LinA-type2.

## Materials and methods

### *In silico* docking

Docking calculations were carried out using GOLD suite 5.1 (Cambridge Crystallographic Data Centre, CCDC) as described earlier (Macwan et al. 2012). The (+) and (–) enantiomers of α-HCH were constructed and subjected to full energy minimization using Insight II 2000.1 builder module (http://www.accelrys.com). The co-ordinates of LinA-type1 (PDB: 3A76) and LinA-type2 (3S5C) were used for docking the substrates. The complexes were evaluated using Goldscore. A maximum of ten models were evaluated with the genetic algorithm method. Default GOLD parameters were used for other values in the calculations. The best predicted substrate-protein complex was analyzed and drawn with PyMOL (The PyMOL Molecular Graphics System, Version 1.7.6.6, Schrödinger, LLC.).

### Generation of mutant LinA enzymes

Genes for the mutants E1–E7 of LinA-type1, which carried one, two, or three of the previously identified changes K20Q, L96C, and A131G (Table 2) were prepared by using variants of LinA-type1 that contained the desired changes as template, and different forward and reverse primers (Table 1) for the different mutants. For example, the gene for the mutant E4 that carries two changes (K20Q and L96C) was constructed in three steps. First, a PCR using the gene *linA*-M9, having the change K20Q, referred here as E1 but generated in an earlier study (Macwan et al. 2011) was used as template, and F1 and R1 as primers (Table 1). The second step was another PCR by using the gene *linA*-M6 (having the changes K20Q, A23G, F68Y, C71T, and L96C), as template, and F1 and R1 as primers (Table 1). In the third step, both the amplified products were digested with Tsp5091, and the resulting 241 bp fragment from the first reaction (representing the 5′ half of *linA*-M9) and 230 bp fragment from the second reaction (representing the 3′ half of *linA*-M6) were joined by ligation. Genes for all the other LinA-type1 mutants were generated likewise, using suitable templates and primer sets, followed with ligation of the desired restriction fragments.

**Table 1:** Primers used in the study

Primer	Primer sequence	Primer details
F1	5′-CATATGAGTGATCTAGACAGACTTGCAA-3′	4–25 nucleotides from 5′ end of *linA*-*type1* preceded by an NdeI site (underlined)
F2	5′-TGGAAGTTCTCTAAGCGCAACGGATGCACG-3′	370–400 nucleotides of *linA*-*type1 *containing DdeI site (underlined) and causes change of residue A131G
F3	3′-TGGAAGTTCTCTAAGCGCAACGGATGCATGAACTATTTC-5′	370–409 nucleotides of *linA*-*type1 *containing DdeI site (underlined) and causes change of residue A131G & T133M
R1	5′-CTCGAGTGCGCCGGACGGTGCGA-3′	448–471 nucleotides of *linA*-*type1* followed by an *Xho*I site (underlined)

All the ligated products i.e., for the mutants E1–E8 were re-amplified by using primers F1 and R1, digested with NdeI and XhoI (whose sites were contributed by the primers), ligated with pET-26b (+) vector (Novagen, Darmstadt, Germany), and cloned in *E. coli* DH5α cells (Macwan et al. 2012). All the constructed genes i.e., for the proteins LinA-type1 (EU863865), -type2 (EU863871) and mutants E1-E8 were cloned and expressed in *E. coli* BL21 (DE3) cells, and the formed proteins were purified by Ni–NTA Superflow column (QIAGEN, Hilden, Germany) at 4 °C, as described earlier (Macwan et al. 2012).

### Chemical synthesis of β-PCCH, γ-PCCH and δ-PCCH

Synthesis of β-PCCH was done according to the method described by Buser and Müller (Buser and Müller 1995) by using 100 mg α-HCH, which was dissolved in 5 ml pyridine and incubated at 65 °C for 14 h. The contents were extracted with *n*-hexane, and washed serially with 5% HCl, 5% NaHCO_3_ and water. The formed β-PCCH was purified by column chromatography, using silica gel 60 (63–100 μm, Merck, Darmstadt, Germany) as stationary phase and *n*-hexane: ethyl acetate (95:5) as mobile phase. The γ- and δ-PCCH were synthesized by a method described by Trantirek et al. (Trantírek et al. 2001). Here, 100 mg γ- or δ-HCH was dissolved in 10 ml acetonitrile, mixed with 5 ml 0.1 M NaOH, and incubated at 40 °C for 20 min. After the reactions, the contents were extracted in *n*-hexane. The formed PCCHs were purified as described for β-PCCH, except that the solvents, *n*-hexane: acetone (98:2) and cyclohexane: acetone (98:2), were used as mobile phase for γ-PCCH and δ-PCCH, respectively.

### HCH-dehydrochlorinase activity

Kinetic parameters for both the wild-type enzyme and the LinA enzyme mutants toward each enantiomer of α-HCH were determined by using purified enzymes. Purities of the enzymes were >95% as judged by densitometry analysis of SDS-PAGE gels. Activities of the different LinA proteins were measured by following utilization of the substrate, as described earlier (Macwan et al. 2012). Briefly, suitable amounts of LinA proteins (0.6 nM for γ-HCH, 3 nM for α-HCH and 6 nM for δ-HCH) were added to the reaction medium of 100 μl containing 50 mM Tris–HCl, pH 8.0, 340 μM HCH isomer or 392 μM PCCH isomer (stock solution 5 mg ml^−1^ in DMSO), and 10% glycerol. The reactions were done in triplicates at 30 °C for different time intervals, and were stopped by acidification to pH < 2.0. Residual HCH was extracted thrice with 1 ml *n*-hexane each time, and suitable aliquots were analyzed by gas chromatography as described before (Macwan et al. 2012). The enantiomer analysis was done by means of an Agilent 7890A gas chromatograph that was equipped with a chiral column (CP-Chirasil-Dex-CB; 25 m × 0.32 mm × 0.25 μm) and an electron capture detector (Zhao et al. 2005). The following temperature program was used: Initial oven temperature at 80 °C (2 min hold), then an increase to 180 °C at a rate of 2 °C min^−1^ with a final hold for 10 min. Injector and detector temperatures were fixed at 250 °C.

### Modeling

**Fig. 2:**
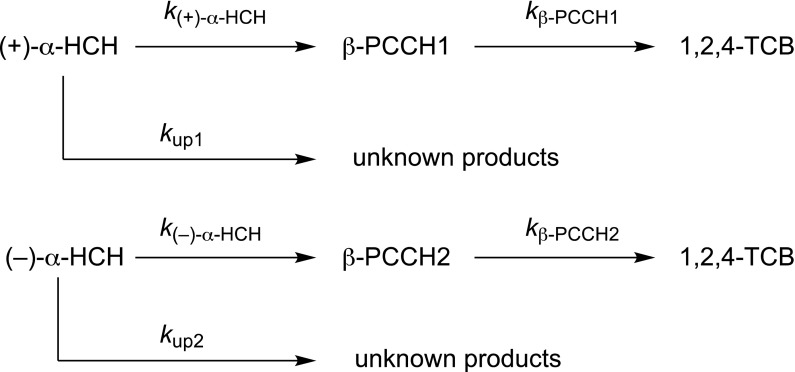
Reaction scheme used for setting up the first-order reaction model with the COPASI software (Hoops et al. 2006) for degradation of α-HCH to β-PCCH

We evaluated the kinetics of HCH dissipation, PCCH formation, and dissipation of PCCH with the modeling software COPASI (Hoops et al. 2006) assuming first-order reactions. The model was set up as shown in Fig. 2. Enantioselectivity (ES) was calculated as follows:

$$ES = \, (k_{\left( + \right) - \alpha - HCH} - k_{{\left( {-} \right) - \alpha - HCH}} )/(k_{\left( + \right) - \alpha - HCH} + k_{{\left( {-} \right) - \alpha - HCH}} )$$

where *k* represents first order rate constants leading to the formation of the respective PCCHs. ES can assume values between –1 and +1. A value of –1 signifies transformation of only the (–) enantiomer, whereas a value of +1 signifies transformation of only the (+) enantiomer.

Values from –1 to +1 indicate a progressive decrease in the preference for the transformation of (–)-α-HCH (or vice versa an increase in the preference for (+)-α-HCH) with a value of zero referring to non-enantioselectivity.

## Results and discussion

### Selection of mutation sites

**Fig. 3:**
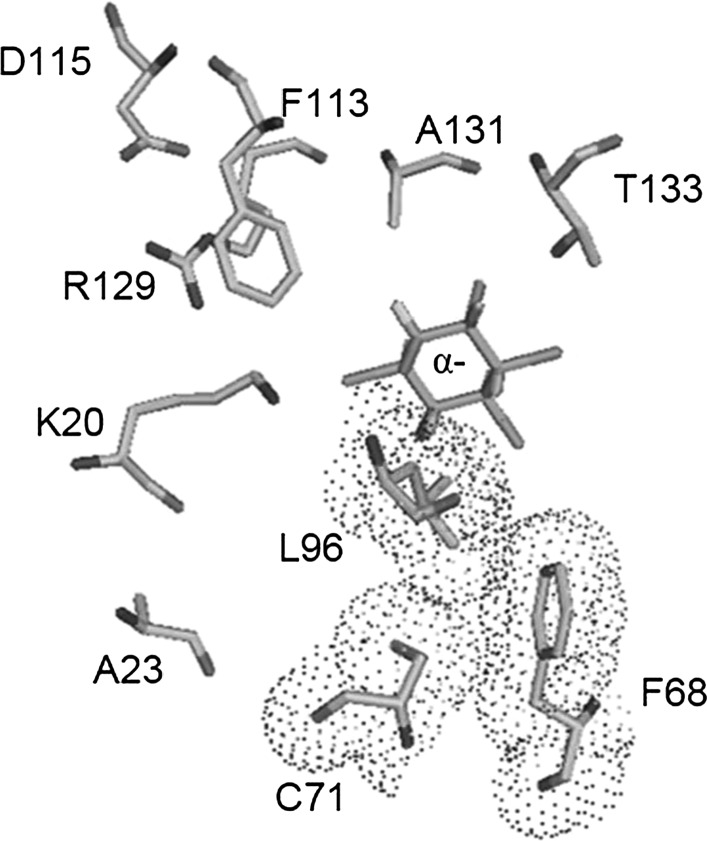
Position of all ten amino acid residues of LinA-type1 that differ from those of LinA-type2 are shown with respect to the best solution docking of (–)-α-HCH (*colored green*) in the active site pocket of LinA-type1

Preliminary analysis (Table S1) of different LinA variants that were generated in an earlier study (Macwan et al. 2012) indicated that three main clusters of residues, i.e., cluster 1 consisting of residues 20 and 23, cluster 2 consisting of residues 68, 71 and 96, and cluster 3 consisting of residues113, 115, 129, 131 and 133 have a role in enantioselectivity. Docking experiments with GOLD Suite showed that the residues 20, 96, 131, and 133 had significant changes in terms of distance of the reactive group of the amino acids to the ligands. In LinA-type1, distances of K20, L96, A131, and T133 from (–)-α-HCH were 3.0, 2.6, 3.2, and 2.7 Å, respectively. They increased to 3.9, 4.6, 4.5 and 2.9 Å, upon change to Q20, C96, G131 and M133, respectively, in LinA-type2 (Figs. 3, S1). Therefore, these residues were chosen for a mutational analysis in which the mutants E1–E8 (Table 2) were constructed and rigorously investigated for enantioselective transformation of HCH isomers and metabolites.

**Table 2:** Mutant LinA-type1 enzymes generated for this study

Mutant enzyme	Amino acid changes
E1	K20Q
E2	L96C
E3	A131G
E4	K20Q, L96C
E5	K20Q, A131G
E6	L96C, A131G
E7	K20Q, L96C, A131G
E8	K20Q, L96C, A131G, T133 M

### Transformation of α-HCH

**Fig. 4:**
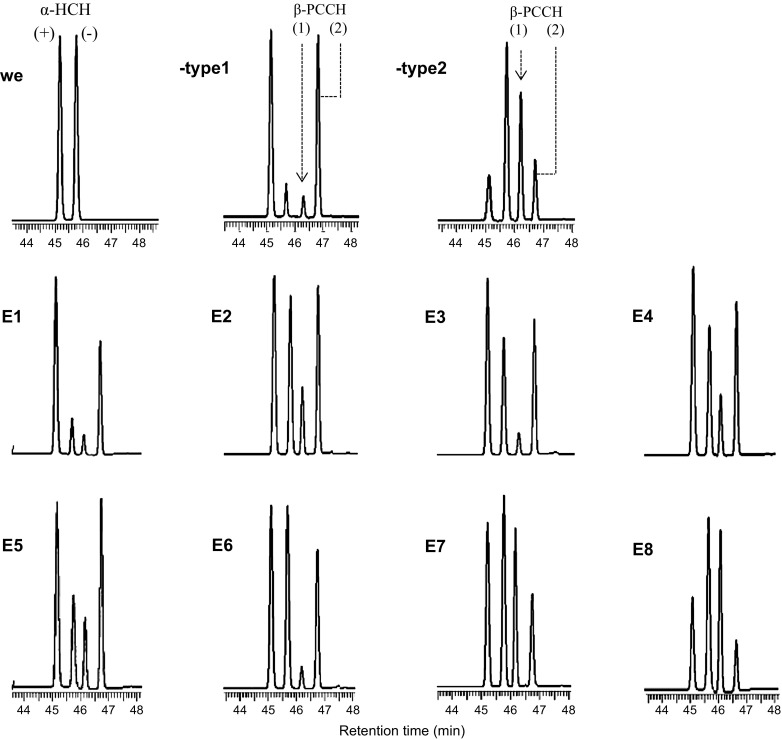
GC-chromatogram depicting enantioselective transformation of α-HCH by LinA-type1, LinA-type2, and eight mutants (E1–E8) after incubations of 60 min. WE represents the racemic starting material. The formed metabolites 1,2,4-trichlorobenzene (TCB) and β-pentachlorocyclohexene enantiomers are also shown. The designations β-PCCH1 and β-PCCH2 designate the enantiomers (3S,4S,5R6R)-1,3,4,5,6-PCCH and (3R,4R,5S,6S)-1,3,4,5,6-PCCH, respectively

**Fig. 5:**
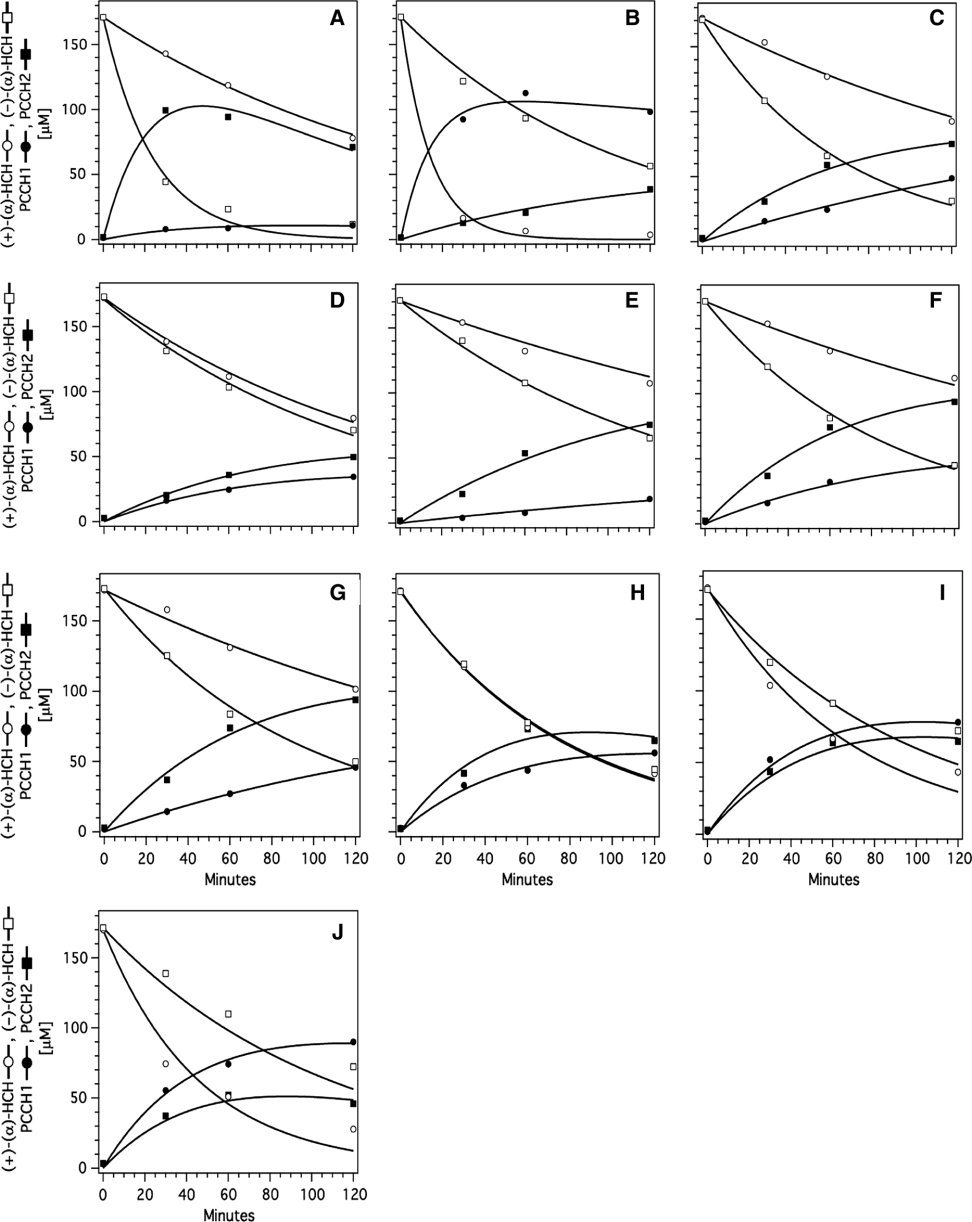
Transformation of α-HCH by LinA-type1, LinA-type2, and various mutant enzymes as monitored by GC-ECD. Continuous lines represent the model fits of the data; corresponding fitted parameters are summarized in Table 3. **A** LinA-type1, **B** LinA-type-2, **C** LinA-E1, **D** LinA-E2, **E** LinA-E3, **F** LinA-E4, **G** LinA-E5, **H** LinA-E6, **I** LinA-E7, **J** LinA-E8

Activities of wild types LinA-type1, LinA-type2, and the mutants E1–E8 were measured. Briefly, all the proteins mediated the metabolism of α-HCH (Table 3) and the reaction in each case was accompanied by the formation of β-PCCH and 1,2,4-TCB (Figs. 4, 5). To close the mass balance, additional reactions leading to unidentified products needed to be included in the fitting procedure (Fig. 2). The rate constants obtained from the best fits of the experimental data (Table 3) showed that, as suggested before (Suar et al. 2005), β-PCCH was indeed the predominant product of α-HCH-transformation by LinA type enzymes.

**Table 3:** Modeled reaction rates, enantioselectivities, and specificity constants of the wild type and mutant LinA proteins with regard to transformation of α-HCH

Enzyme/LinA mutant	*k*_(+)-α-HCH_ [×10^−3^ min^−1^]	*k*_up1_ [×10^−3^ min^−1^]	*k*_(–)-α-HCH_ [×10^−3 ^min^−1^]	*k*_up2_ [×10^−3 ^min^−1^]	ES	*k*_cat_/*K*_m _(+)-α-HCH [s^−1^ M^−1^]	*k*_cat_/*K*_m_ (–)-α-HCH [s^−1^ M^−1^]
-type1	2 ± 0.2	4.3 ± 0.8	38 ± 2.9	4.1 ± 3.4	−0.9 ± 0.012	113.0	2245.7
-type 2	46 ± 3.8	24 ± 4.0	3 ± 0.2	6.5 ± 0.6	0.88 ± 0.012	2771.6	178.3
E1 (Q)	3 ± 0.2	1.8 ± 0.4	8 ± 0.4	6.7 ± 0.7	−0.47 ± 0.032	181.0	500.2
E2 (C)	3.6 ± 0.2	3.1 ± 0.4	5 ± 0.3	3.2 ± 0.5	−0.15 ± 0.036	214.4	291.7
E3 (G)	1 ± 0.1	2.4 ± 0.6	6 ± 0.6	1.5 ± 0.9	−0.71 ± 0.038	61.7	367.4
E4 (QC)	3.9 ± 0.2	0.0 ± 0.4	10 ± 0.5	1.6 ± 0.7	−0.44 ± 0.031	234.2	597.3
E5 (QG)	3.1 ± 0.2	1.2 ± 0.3	1 ± 0.5	1.1 ± 0.6	−0.53 ± 0.027	182.8	592.2
E6 (CG)	7.6 ± 0.5	5.3 ± 0.9	13 ± 0.6	0.0 ± 0.2	−0.25 ± 0.037	454.6	753.7
E7 (QCG)	12.5 ± 1.0	2.1 ± 1.4	10 ± 0.9	0.0 ± 1.3	0.09 ± 0.058	745.1	622.9
E8 (QCGM)	14.7 ± 1.3	7.2 ± 1.9	9 ± 0.9	0.0 ± 1.3	0.23 ± 0.062	874.4	552.1

The wild type enzymes, LinA-type1 and LinA-type2, exhibited strong opposite enantioselectivity for the (–) and (+) enantiomer of α-HCH (Figs. 4, 5) and ES values for the two reactions were −0.90 and 0.88, respectively (Table 3). The analysis revealed that the specificity constant (*k*_cat_/*K*_m_) of LinA-type1 for the metabolism of (–) enantiomer of α-HCH was 24-fold higher than that of LinA-type2 (Table 3). Conversely, the specificity constant (*k*_cat_/*K*_m_) of LinA-type2 for the metabolism of (+) enantiomer of α-HCH was 12-fold higher than that of LinA-type1 (Table 3). The specificity constants (*k*_cat_/*K*_m_) of the mutants E1, E2, E3, E4, and E5 for the (–) enantiomer were reduced 3–8 fold, when compared to that of wild type LinA-type1, but were in the range (within two folds) of that for the (+) enantiomer.

The enantioselectivity values (ES values) of the mutants E1, E2, and E3 that differed from LinA-type1 by one residue, namely K20Q, L96C, and A131G, respectively, or the mutants E4 or E5 that were carrying two changes, namely K20Q, L96C and K20Q, A131G, respectively, were less negative than that of the wild type linA-type1, but preferential metabolism of the (–) α-HCH was still observed with all these mutants (Fig. 5). However, the substantially less negative ES values (Table 3) suggest that their preference for the transformation of the (–) enantiomer was lower than that of LinA-type1. Furthermore, an ES value of −0.25 for the mutant E6 implies that enantioselectivity towards the (–) enantiomer was further diminished in this mutant. It needs to be noted that the ES values refer to the rate constants leading to β-PCCH only. In the case of mutant E6, simultaneous disappearance of both enantiomers might suggest non-enantioselectivity, whereas selective formation of β-PCCHs indicates enantioselectivity (Fig. 5).

Interestingly, a slight preferential metabolism of the (+) enantiomer over the (–) enantiomer, in other words reversed enantioselectivity, was observed for the mutant E7 carrying the three changes K20Q, L96C, and A131G (Figs. 4, 5). The preference, however, was much less pronounced than that of LinA-type2 (Table 3), suggesting that additional divergent amino acids in LinA-type2 are responsible for further enhancing enantioselectivity. Indeed, reversed enantioselectivity was much further enhanced in the mutant E8, which carried the additional change T133M (Table 3; Figs. 4, 5). These results show that not one single divergent amino acid determined enantioselectivity, but that the cumulative effect of the four diverging residues 20 (K, C) 96 (L, C), 131 (A, G), and 133 (T, M) was needed for the opposite enantioselectivity of LinA-type1 and LinA-type2 with regard to the transformation of α-HCH.

Enantioselective transformation i.e., preferential metabolism of (–)-α-HCH by LinA-type1 and of (+)-α-HCH by LinA1 was described nearly ten years ago (Suar et al. 2005), but the molecular features responsible for this property have not been understood till date. In this work, we describe that the amino acids K20, L96, A131, and T133 are key determinants for the observed enantioselectivity of LinA-type1. These, when changed to Q20, C96, G131, and M133 as present in LinA1 and LinA-type2, caused a reversal in the enantioselectivity, leading to the preferential metabolism of (+)-α-HCH (Fig. 5). However, complete reversal of enantioselectivity was not achieved and, therefore, additional amino acid residues further away from the active site might also be involved in enhancing enantioselectivity.

### Transformation of γ- and δ-HCH

Besides α-HCH, all LinA variants tested i.e. LinA-type1, LinA-type2, and the mutants E1 to E8 also transformed γ-HCH and δ-HCH to γ-PCCH and δ-PCCH, respectively, and ultimately to TCBs. Enantiomer analysis revealed that incubations of LinA-type1 with γ-HCH led to the formation of only γ-PCCH-2 (Fig. S2), whereas incubations with LinA-type2 led to the formation of a mixture that contained nearly equal amounts of γ-PCCH-1 and γ-PCCH-2. All the LinA-type1 mutants *i.e.* E1–E8 led to the formation of γ-PCCH-2 as the major enantiomer (Fig. S2). Similar results were obtained for the transformation of δ-HCH. While δ-PCCH-2 was the major product formed by LinA-type1, a mixture containing nearly equal amounts of δ-PCCH-1 and δ-PCCH-2 was formed by LinA-type2. No change in the enantioselectivity of the mutants E1–E6 was observed and δ-PCCH-2 was still the major metabolite formed. The mutants E7 and E8, however, caused the formation of a mixture of δ-PCCH-1 and δ-PCCH-2, as formed by LinA-type2 (Fig. S2). These results suggest that the targeted amino acids in most mutants, except those in mutant E7 and E8 with regard to transformation of δ-HCH, did not play a critical role in determining enantioselectivity of the transformation of γ-HCH or δ-HCH by LinA-type1.

### Transformation of β-, γ- and δ-PCCH

All the LinA proteins mediated the metabolism of β-, γ- and δ-PCCH, formed after the metabolism of α-, γ- and δ-HCH, respectively, and the reactions were accompanied with the formation of TCBs (Fig. S3 and S4). LinA-type1 exhibited enantioselectivity towards the metabolism of β-PCCH-1, but LinA-type2 used β-PCCH-2 preferentially (Fig. S3). The enantioselectivity of the mutants E1–E7 was identical to the one of LinA-type1 and preferential metabolism of β-PCCH-1 was observed. The enantioselectivity of the mutant E8, however, was reversed and preferential metabolism of β-PCCH-2 was observed (Fig. S3). In the case of γ-PCCH, LinA-type1 and LinA-type2 exhibited preference for the metabolism of γ-PCCH-2 and γ-PCCH-1, respectively, and the reaction was accompanied with the formation of a mixture of 1,2,3- and 1,2,4-TCB in both cases (Fig. S4). Similar to LinA-type1, the mutants E1–E3 also exhibited preference for the metabolism of γ-PCCH-2. In the mutants E4–E7, however, the preference was reversed, and higher turn over of γ-PCCH-1 was observed. The preference for γ-PCCH-1 was further enhanced in the mutant E8 (Fig. S4). Similar to β- and γ-PCCH, enantioselectivity was also observed during the metabolism of δ-PCCH. While LinA-type1 exhibited preference for δ-PCCH-2, LinA-type2 had preference for δ-PCCH-1 (Fig. S4). The enantioselectivity of the mutants E1, E5, and E7 was the same as for LinA-type1, and preferential metabolism of δ-PCCH-2 was observed. In the mutants E2, E3, E4, and E6 enantioselectivity was lost and δ-PCCH-1 and δ-PCCH-2 were turned over equally. The enantioselectivity of the mutant E8, however, was reversed and preferential metabolism of δ-PCCH-2 was observed (Fig. S4). The observed enantioselective transformation by LinA-type1 and LinA-type2 proteins of different PCCHs is in agreement with earlier studies made on β-PCCH (Suar et al. 2005), γ-PCCH (Trantírek et al. 2001) and δ-PCCH (Geueke et al. 2013). Change in the enantioselectivity of LinA-type1 upon the change in four amino acid residues, as present in mutant E8, suggests that these amino acids also play a role in the enantioselectivity of LinA enzymes for differ PCCHs.

Overall, the residues K20, L96, A131, and T133 appear to be important determinants for the enantioselectivity of LinA proteins towards the transformation of all the studied substrates, except γ-HCH. Amino acids responsible for the enantioselective transformation of γ-HCH might be different, and need to be identified in further studies.

## Electronic supplementary material

Below is the link to the electronic supplementary material.

Supplementary material 1 (PDF 625 kb)
