# Supplementary material for: Important amino acid residues of hexachlorocyclohexane dehydrochlorinases (LinA) for enantioselective transformation of hexachlorocyclohexane isomers
Source: Biodegradation. 2017 Mar 1;28(2):171–80. doi: 10.1007/s10532-017-9786-9 (PMC5422493; doi:10.1007/s10532-017-9786-9)
Supplement: Supplementary file 1 — Supplementary material 1 (PDF 625 kb) [file 10532_2017_9786_MOESM1_ESM.pdf]

Supplemental Material for:

Important Amino Acid Residues of Hexachlorocyclohexane Dehydrochlorinases (LinA) for Enantioselective Transformation of Hexachlorocyclohexane Isomers

Nidhi Shrivastava[a,b], Ankit S. Macwan[c], Hans-Peter E. Kohler[d], and Ashwani Kumar[a,b#]

[a]Academy of Scientific and Innovative Research, New Delhi, India; [b]Environmental Biotechnology Section, CSIR-Indian Institute of Toxicology Research, Lucknow, India; [c]Department of Clinical and Experimental Medicine, Linköping University, Linköping, Sweden; [d]Department of Environmental Microbiology, Swiss Federal Institute for Aquatic Science and Technology (Eawag), Dübendorf, Switzerland.

Keywords: HCH Dehydrochlorinase LinA, Enantioselectivity, α-HCH enantiomers

#Adress correspondence to Hans-Peter E. Kohler at <hanspeter.kohler@eawag.ch>

Preliminary enantioselectivity analysis. Enantioselectivity analysis of different LinA variants with one or more of the ten residue changes found between LinA-type1 and -type2 indicated the contribution of individual residues. Based on the selective degradation of each enantiomer by individual variants (Table S1), it can be anticipated that three main clusters of residues have role in enantioselectivity, *i.e*. cluster 1 consisting of residues 20 and 23, cluster 2 consisting of 68, 71 and 96 and rest of the residues in cluster 3 consisting 113, 115, 129, 131 and 133. Generation of mutants M1 to M8 are described in (Macwan et al. 2012).

Table S1: Enantioselectivity of different LinA variants

**LinA variant**	**Amino acid position**										**Activity** (after 10 min at 30°C)	**α-HCH** (+) : (-)
	20	23	68	71	96	113	115	129	131	133		
LinA-type1	K	A	F	C	L	F	D	R	A	T	95%	72:28
LinA-type2	Q	G	Y	T	C	Y	N	L	G	M	100%	25:75
M1	-	-	Y	T	C	Y	N	L	G	M	35%	39:61
M2	-	-	-	-	C	Y	N	L	G	M	35%	49:51
M3	-	-	-	-	-	Y	N	L	G	M	Nil	-----
M4	-	-	-	-	-	-	-	L	G	M	Nil	-----
M5	Q	G	Y	T	C	Y	N	-	-	-	40%	44:56
M6	Q	G	Y	T	C	-	-	-	-	-	32%	45:55
M7	Q	G	Y	T	-	-	-	-	-	-	56%	70:30
M8	Q	G	-	-	-	-	-	-	-	-	80%	63:37

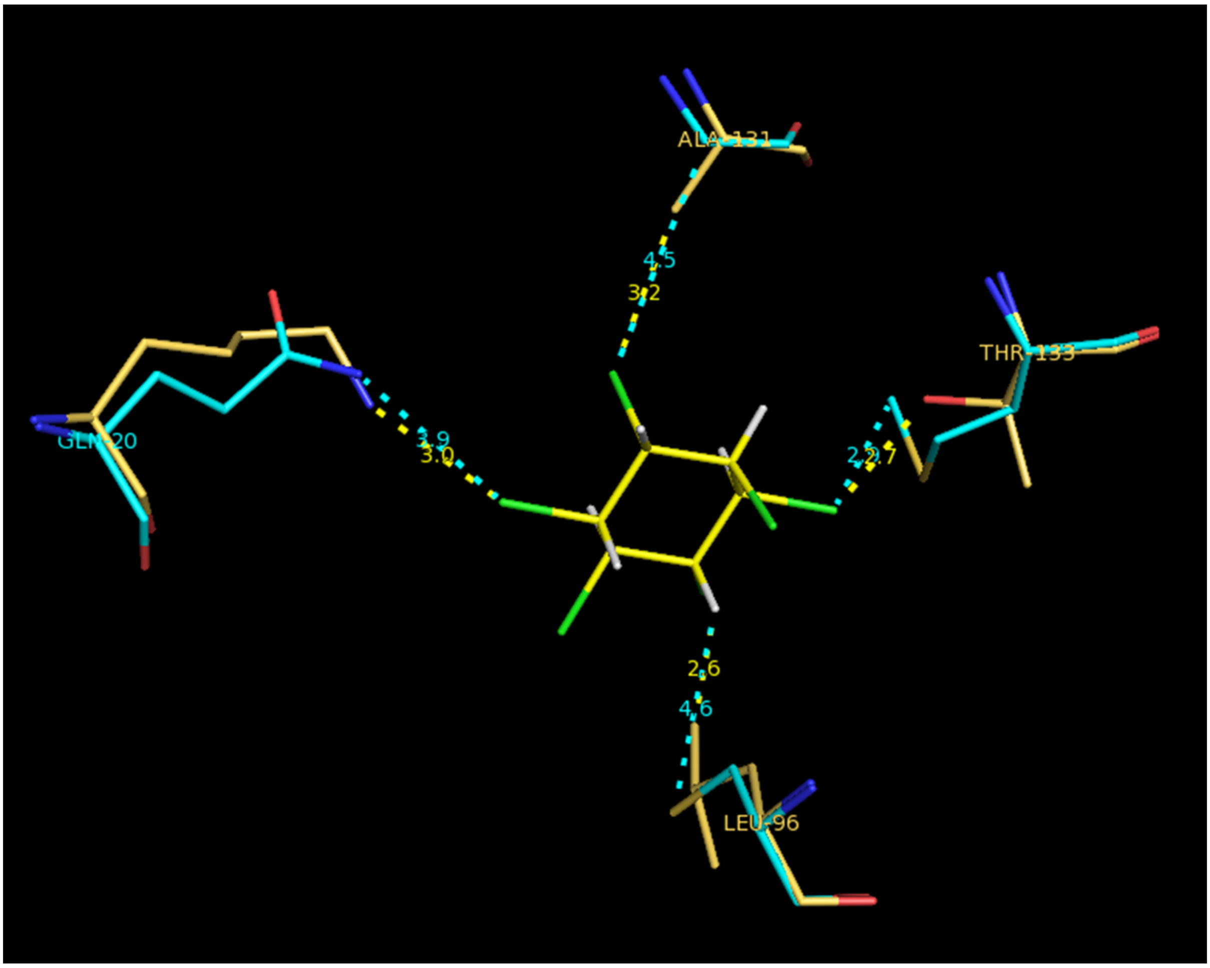

Fig. S1. Orientation and position of residues 20, 96, 131, and 133 of LinA-type1 (golden) and of LinA-type2 (blue) are shown with respect to the best solution docking of (–)-α-HCH in the active site pocket of LinA-type 1.

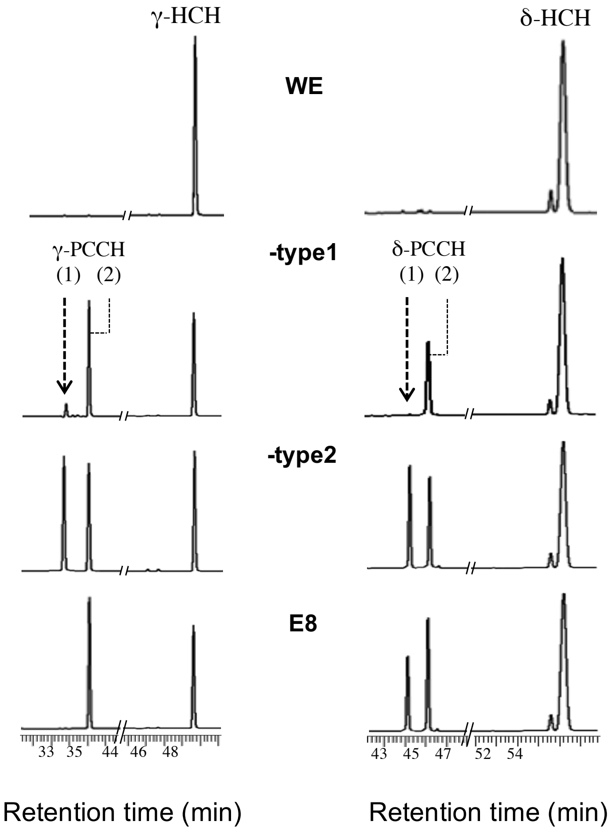

Fig. S2. GC-chromatogram depicting transformation of γ-HCH (30 min incubation) and δ-HCH (60 min Incubation) by LinA-type1, LinA-type2, and by mutant E8. WE represents the starting material. The formed metabolites 1,2,3- and 1,2,4-trichlorobenzene (TCB) and γ-pentachlorocyclohexene enantiomers (γ-PCCH-1 and γ-PCCH-2), as well as δ-pentachlorocyclohexene enantiomers (δ-PCCH-1 and δ-PCCH-2) are also shown. Reactions of mutants E1 to E7 with γ-HCH, being similar to LinA-type1, are not shown. Likewise, reactions of mutants E1 to E7 with δ-HCH are not shown, as those of E1 to E6 were similar to LinA-type1, and of E7 to E8. The designations γ-PCCH-1 and γ-PCCH-2 have been used for the enantiomers (3*S*,4*R*,5*R*,6*S*)-1,3,4,5,6-PCCH and (3*R*,4*S*,5*S*,6*R*)-1,3,4,5,6-PCCH, respectively, as described in the text. The stereochemistry of δ-PCCH-1 and δ-PCCH-2, however, is not yet known.

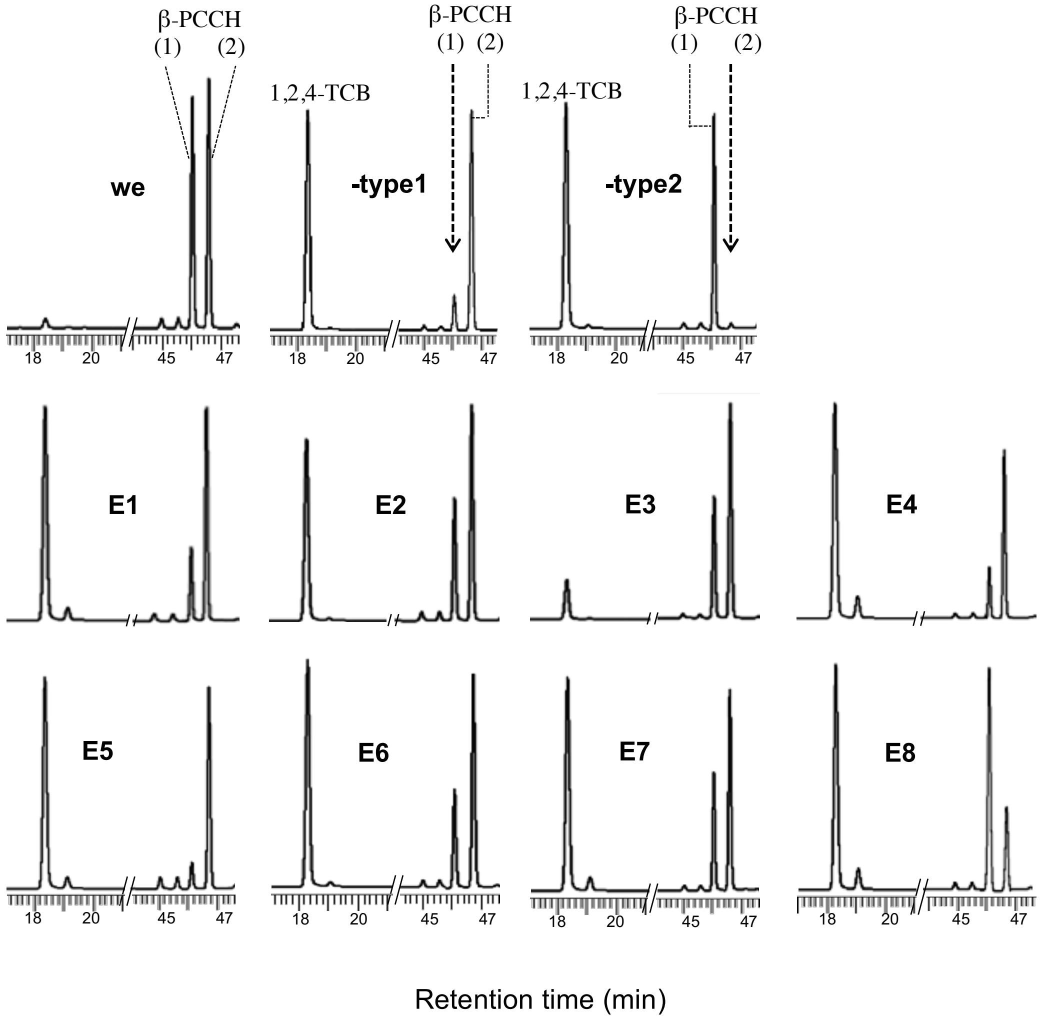

Fig. S3. GC-chromatogram depicting enantioselective transformation of β-PCCH (90 min incubation) by LinA-type1, LinA-type2, and eight mutants (E1 to E8). WE represents the racemic starting material. The formed metabolite 1,2,4-trichlorobenzene (TCB) is also shown.

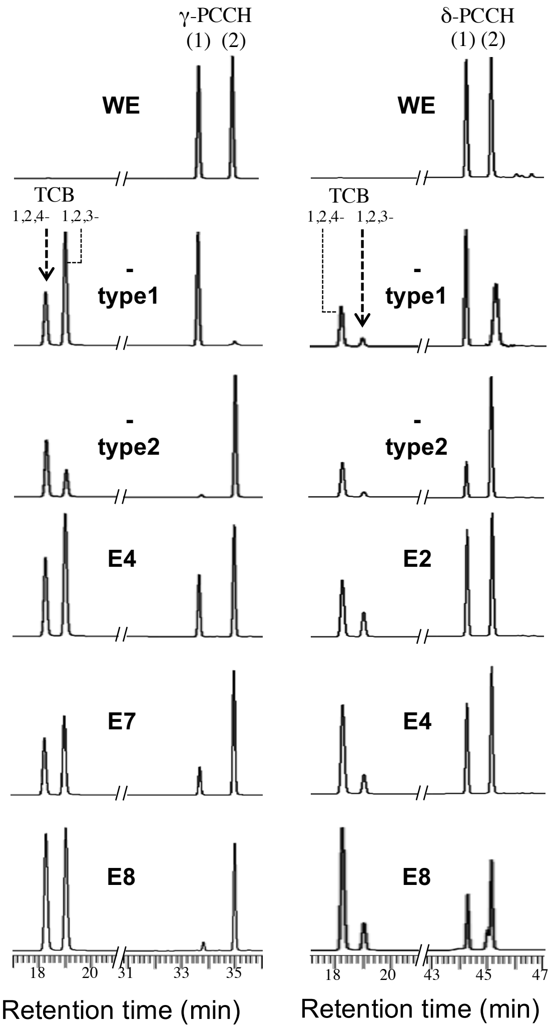

Fig. S4. GC-chromatogram depicting transformation of γ-PCCH and δ-PCCH (90 min incubation) by LinA-type1, -type2, and by various mutants (described in Table1). WE represents the racemic starting material. The formed metabolites 1,2,3- and 1,2,4-trichlorobenzene (TCB) are also shown. Reactions of mutants E1 to E3, E5, and E6 with γ-PCCH are not shown (being similar to LinA-type1 and E4, respectively). Likewise, reactions of mutants E1, E5, and E7 with δ-PCCH were similar to LinA-type1, and of E3 and E6 to E8.

References:

Macwan AS, Kukshal V, Srivastava N, Javed S, Kumar A, Ramachandran R (2012) Crystal Structure of the Hexachlorocyclohexane Dehydrochlorinase (LinA-Type2): Mutational Analysis, Thermostability and Enantioselectivity Plos One 7 doi:10.1371/journal.pone.0050373
